# Direct current-tunable MHz to multi-GHz skyrmion generation and control

**DOI:** 10.1038/s41598-019-45972-9

**Published:** 2019-07-01

**Authors:** Arash Mousavi Cheghabouri, Mehmet C. Onbasli

**Affiliations:** Department of Electrical and Electronics Engineering, Koç University, Sarıyer 34450 Istanbul, Turkey

**Keywords:** Magnetic devices, Electronic and spintronic devices

## Abstract

Skyrmions offer high density, low power, and nonvolatile memory functionalities due to their nanoscale and topologically-protected chiral spin structures. For integrated high-bandwidth devices, one needs to control skyrmion generation and propagation rates using current. Here, we introduce a skyrmion initialization and control method to generate periodic skyrmions from 114 MHz to 21 GHz using spin-polarized direct current. We first initialize a stable magnetic domain profile that is pinned between a notch and a rectangular constriction using a DC pulse. Next, we pass spin-polarized DC charge current to eject periodic skyrmions at a desired frequency. By changing the DC current density, we demonstrate in micromagnetic simulations that skyrmion generation frequencies can be controlled reversibly over more than seven octaves of frequencies. By using domain pinning and current-driven skyrmion motion, we demonstrate a highly tunable and DC-controlled skyrmion signal source, which pave the way towards ultra wideband, compact and integrated skyrmionic circuits.

## Introduction

Magnetic skyrmions are topologically-protected chiral spin structures that retain topological charge^1^. In many cases, skyrmions arise from the competition between Dzyaloshinskii-Moriya interaction (DMI) and perpendicular axis energy interactions such as Heisenberg exchange or a combination of various uniaxial anisotropy energy terms^2–4^ or dipolar interaction in magnetic materials^5,6^. Néel or Bloch types of skyrmions may emerge^7,8^ depending on the nature of DMI in materials with perpendicular magnetic anisotropy. Skyrmions and chiral structures have been proposed also for in-plane magnets^9^. Chiral Skyrmions were first observed as lattices in non-centrosymmetric magnetic crystals like MnSi^10–13^. Single skyrmions were later realized in bilayers and multilayers of magnetic materials and heavy metals^14,15^. Research on skyrmions is intensive due to their numerous attractive properties including nanometer diameters for high-density storage, room temperature stability^16–19^, current-controlled motion^20^ low power translational movement^21,22^, topological charge and symmetry protection against large defects^23,24^, detectability by various methods such as magnetic force microscopy^25^ and soft x-ray scattering^26,27^, detection by Lorentz transmission microscopy^13,28^, room temperature electrical readout using topological Hall effect, and electrical nucleation and stabilization. Each of these demonstrations with the elimination of skyrmion Hall effect^29–33^ help lay the foundations for skyrmion-based racetrack skyrmion devices as a promising platform for the beyond Moore technologies^34^.

For capturing the advantages of skyrmions and using them as signal carriers, key skyrmionic components such as skyrmion signal generator, encoder, splitter, combiner, detector and fundamental logic elements must be constructed. One of the most important elements in skyrmionics is a generator that can initialize, stabilize and generate skyrmions at desired frequencies. Several researchers have proposed and demonstrated skyrmion nucleation schemes. Earlier demonstrations include skyrmion generation from the edge of a notch by spin transfer torques^35^, vertical spin current injection^23,36,37^, electric field pulses^38^, blowing skyrmion bubbles with spin polarized current through a constriction^39,40^, bending a domain wall (DW) in the exit of a geometric constriction^41^, current driven nucleation in bilayers^42^, and magnetic field domain nucleation and conversion into skyrmions^43^. In metallic magnetic films, skyrmions have been created, switched^44^, stabilized^30^, dynamically stabilized in absence of DMI^45^ or stabilizing magnetic field^46^, deleted^47^, merged, duplicated^48^, and driven by current^16^. Skyrmions move across a chip by spin transfer torque exerted from an applied spin-polarized current^21,23,35,49^. These spin transfer torques can be applied either by a direct spin-polarized current through the ferromagnetic film or by injecting a vertical spin current such as spin Hall currents injected from a heavy metal layer^50^. The ability to move skyrmions by spin currents paves the way of designing skyrmion-based racetrack devices, which can convert DWs into skyrmions and vice versa. Controlling the skyrmions in a racetrack device paves the way to skyrmions logic gates. Although a universal skyrmion logic gate set is yet to be demonstrated, skyrmionic AND and OR gates have been proposed theoretically^48^.

While these demonstrations lay the foundations, a current-driven and wideband skyrmion generator is missing. Racetrack skyrmion generators were previously demonstrated^39,40^ or proposed^51^. Nevertheless, the controllability issues, large footprint, low frequency, external magnetic field or microwave dependence of these skyrmion generators make them less ideal for logic or memory applications. Here, we introduce in computational micromagnetic models, a novel skyrmion generator by combining DW pinning and DW-to-skyrmion conversion to generate skyrmions of a desired frequency using spin-polarized DC charge currents in absence of external magnetic fields. We first initialize a skyrmion and drive the skyrmion between a pinning notch and a pinning wall to obtain a stable and expanded magnetic domain. Next, we apply spin-polarized charge current across the domain profile to pinch skyrmions out of an expanded magnetic domain. We then demonstrate that by tuning the applied current density, the skyrmion generation rate can be controlled reversibly in absence of external magnetic field.

## Magnetic Domain Expansion

Previous research on DW motion revealed^52^ many interesting properties of DWs in nanoscale including DW pinning by indentations, notches, and kinks. These studies found that notches and geometric defects can pin a DW. The DW chirality affected the depinning energy. This brought up opportunities for expanding a magnetic domain, which were used to electrically switch magnetic materials. In this study, we use the magnetic domain expansion to generate skyrmions.

The presence of the notch in a channel brings an energy barrier that pins the magnetic DWs. Depinning current density is the minimum current density required for the DW to overcome a notch. In this part, we investigate the depinning current densities for the left and right DWs for an isolated notch. With our configuration, the depinning current density for the right DW j_R_ is smaller than that of the left DW j_L_. The spin-polarized current pushes the DWs to the right. When the applied current is lower than j_R_, the pinning potential of the notch prevents the right DW from passing over the notch. With the right DW pinned to the notch and the left DW pushed to the right by the current, the domain gets smaller and eventually vanishes (Supplementary Movie S1). Currents greater than j_L_ are large enough to push both walls beyond the notch, and the magnetic domain passes the notch completely (Supplementary Movie S2). With a current between j_R_ and j_L_ the right DW passes the notch while the left DW is pinned. This results in an expanding magnetic domain (Supplementary Movie S3). For the presented parameters and geometry, j_R_ = 1.10 × 10^12^ A⋅m^−2^ and j_L_ = 1.72 × 10^12^ A⋅m^−2^.

Skyrmions, similar to magnetic DWs, move along the direction of charge carriers. We use current to push a skyrmion into a channel. It was shown before that pushing a skyrmion into a channel narrower than the skyrmion diameter breaks the topological protection of the skyrmion and results in transforming the skyrmion into a magnetic domain^43^. This magnetic domain is then expanded to generate skyrmion using a special geometry. The types of the DWs determine the skyrmion type. If the skyrmion is Néel (Bloch), the resultant DWs will be Néel (Bloch).

### Periodic skyrmion generation

**Figure 1:**
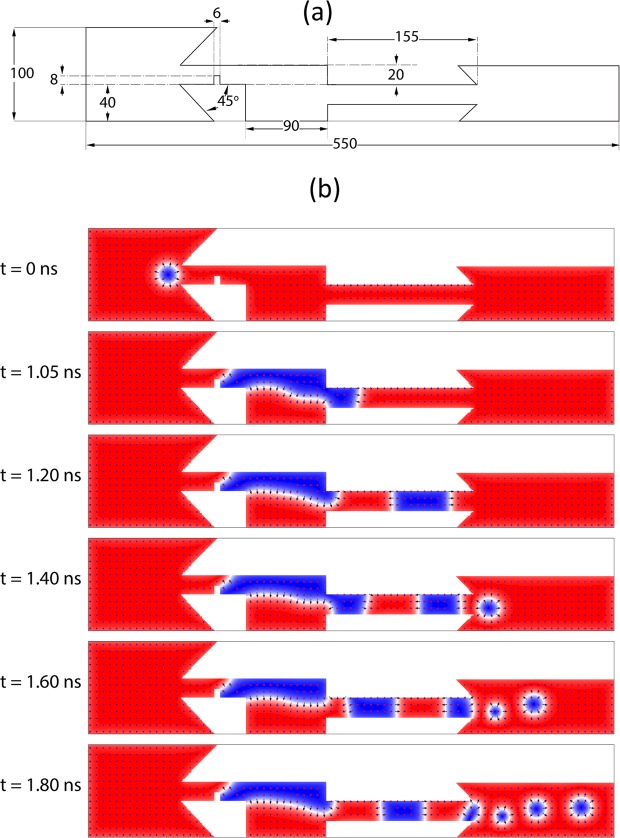
Device dimensions and Néel skyrmion generation in regular operation regime. (**a**) Device dimensions and labels, all annotations are in nanometers. (**b**) Skyrmion generation with notch pinning of the DW. In this case the current lies between ${{\rm{j}}}_{{\rm{R}}}=1.32\times {10}^{12}\,{\rm{A}}\cdot {{\rm{m}}}^{-2}$ and ${{\rm{j}}}_{{\rm{W}}}=1.73\times {10}^{12}{\rm{A}}\cdot {{\rm{m}}}^{-2}$. The periodic skyrmions are generated purely by DC in absence of external magnetic fields.

Figure 1(a) shows the designed geometry of the device and the skyrmion generation process. The structure consists of the following parts from left to right: Input track, the notch channel, reservoir, output channel and the output track.

The process of skyrmion generation starts with the stabilization of a skyrmion at the input track. We apply a spin-polarized direct current with density j = 1.4 × 10^12^ A⋅m^−2^ and polarization P = 1 towards the left side of the generator. Thus, the electrons move from left to right and push the DWs and the skyrmions from left to right. This current is large enough to push the skyrmion into the notch channel and break the topological protection of the skyrmion. The skyrmion converts into a magnetic domain with two walls with opposite chiralities and different depinning currents. The depinning current of the left DW is bigger than that of the right DW. The applied current is between the depinning currents of the left and right DWs.

With the left DW pinned and the right DW pushed to the right, the magnetic domain expands until the domain reaches to the right wall of the reservoir. At this stage, horizontal domain expansion is inhibited and the magnetic domain expands in transverse direction. This transverse expansion results in a magnetic domain at the entrance of the output channel. This behavior leads the current to push the domain into the output channel and pinch out a separate domain into the output channel from the main domain in the reservoir. This separated domain moves along the output to the end of the channel with current while the main domain keeps expanding again. Pushing this magnetic domain out in the output track results in the DW to skyrmion conversion and formation of a skyrmion at the output track^43^. The process of expansion and pinching of the magnetic domain continues if the DC is applied. This process results in the periodic generation of skyrmions. The spin-polarized current continues to push the magnetic domain into the wider output area to generate a skyrmion from the domain (Supplementary Movie S5). The process is illustrated in Fig. 1(b).

### Different operation regimes of the tunable skyrmion generator

The geometric complexity of the device brings about different energy barriers, which play various roles to produce multiple operational regimes.

**Figure 2:**
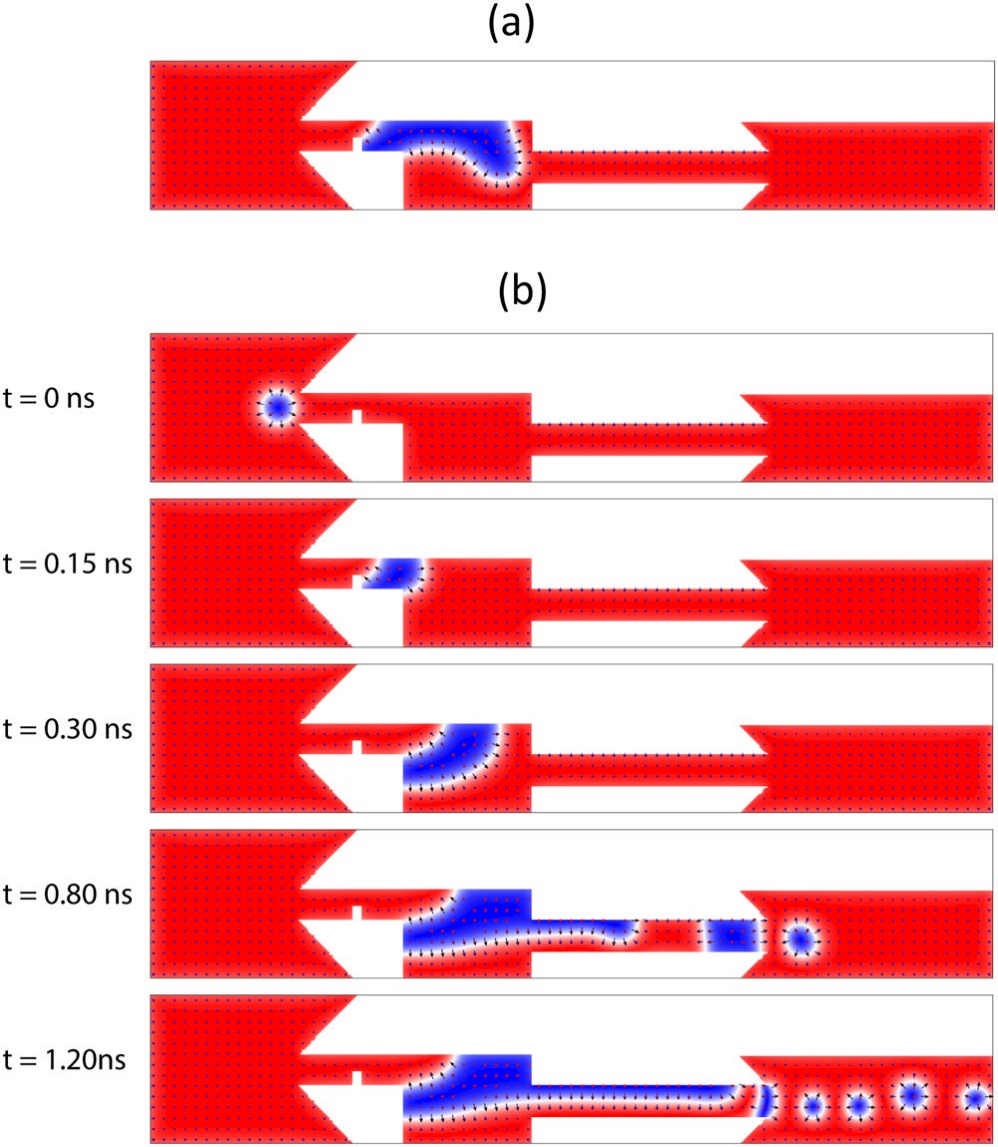
Operation regimes driven by lower and higher currents. (**a**) When the current is less than ${{\rm{j}}}_{{\rm{R}}}=1.32\times {10}^{12}\,{\rm{A}}\cdot {{\rm{m}}}^{-2}$ the magnetic domain stops and will not expand. (**b**) For currents greater than ${{\rm{j}}}_{{\rm{W}}}=1.80\times {10}^{12}{\rm{A}}\,\cdot {{\rm{m}}}^{-2}$ the magnetic domain depins from the notch and pins on the left wall of the reservoir. Each case works in absence of external magnetic field.

The right wall of the reservoir induces an energy barrier (or a repelling force) on the magnetic DW. For the domain to overcome this force and reach the right wall of the reservoir, the current density must be greater than j_Rw_ = 1.32 × 10^12^ A⋅m^−2^. For currents smaller than this, the magnetic domain stops extending before reaching to the right wall of the reservoir (Supplementary Movie S4). Figure 2(a) shows this behavior for j = 1.30 × 10^12^ A⋅m^−2^.

The geometric indentation of the junction of the reservoir and the notch channel acts as an anti-notch and has its own pinning potential. If the current is large enough to depin the left DW from the notch, it pins to left DW of the reservoir.

Figure 2(b) shows the situation for currents larger than the left DW depinning current of the notch j_L_. The magnetic DW depins from the notch, but pins to the left wall of the reservoir. The skyrmion generation does not stop (Supplementary Movie S6). This state is stable until the current reaches j_W_ = 2.73 × 10^12^ A⋅m^−2^. This current density depins the domain and the magnetic domain moves to the right and vanishes after reaching the right wall of the reservoir (Supplementary Movie S7).

We can summarize the working principles of the device as follows:

1. The notch pins the left DW.
2. The spin-polarized direct current expands the magnetic domain.
3. The domain stops at the right wall of the reservoir
4. The domain expands in the transverse direction.
5. The domain reaches the output channel.
6. DC pinches out and pushes a part of the domain into a new domain in the output channel.
7. The current pushes the separated domain in the output channel to the output track and the domain transforms to a skyrmion.

Steps 4 to 7 happen repeatedly and lead to periodic skyrmions. The skyrmion generation frequency depends on the timing of these steps. This timing depends on the applied current, material properties (Gilbert damping, DMI constants, exchange and other anisotropy terms, saturation magnetic moment) and geometric properties. In this study, we investigate the effects of applied current density and the damping coefficient. We also vary the thickness of the material (Cobalt layer thickness from 1 to 4 nm) to investigate that the device is robust and works with slightly shifted frequencies.

### Alternative operation regimes

Although the device is initialized with a skyrmion, since the domain expansion starts with a magnetic domain, the device may be started with a magnetic domain as well. This case is demonstrated in supplementary materials and movie (Supplementary Movie S9). As shown in Fig. 2, the magnetic domain may be pinned to the reservoir wall too and the notch is not necessary. The domain could also be pinned to the reservoir without passing the small notch. This situation for both starting with skyrmion and domain wall is demonstrated in the supplementary materials and movies (Supplementary Movies S10 and S11). The 45° vertices of the device may also be turned into rectangular vertices in favor of simplicity of fabrication (Supplementary Movie S12). These simplifications do not result in the failure of the device, although the device operates in a narrower current range.

### A note on bulk DMI interaction

All the simulations are carried out for the bulk interaction and Bloch skyrmions too. The results are identical to the presented interface interaction and Neel skyrmions. A section in the supplementary materials shows the results for the bulk interaction and Bloch type skyrmions. Supplementary Movie 13 shows one simulation for the Bloch type skyrmions.

## Results

Based on the dominant DMI coefficient (bulk or interface) of the medium, there are two types of skyrmions. Bloch and Néel skyrmions are associated with bulk and interface DMI interactions, respectively. Both types of skyrmions can transform into magnetic domains when pushed into a channel narrower than the skyrmion diameter. We calculated the stability, skyrmion generation rate and the other parametric modeling for the Bloch skyrmions with bulk interaction. These simulation results are identical to those of the Néel skyrmions with interface interaction. Here, we only present the results of interface DMI with Néel skyrmions.

### Skyrmion generation frequency

**Figure 3:**
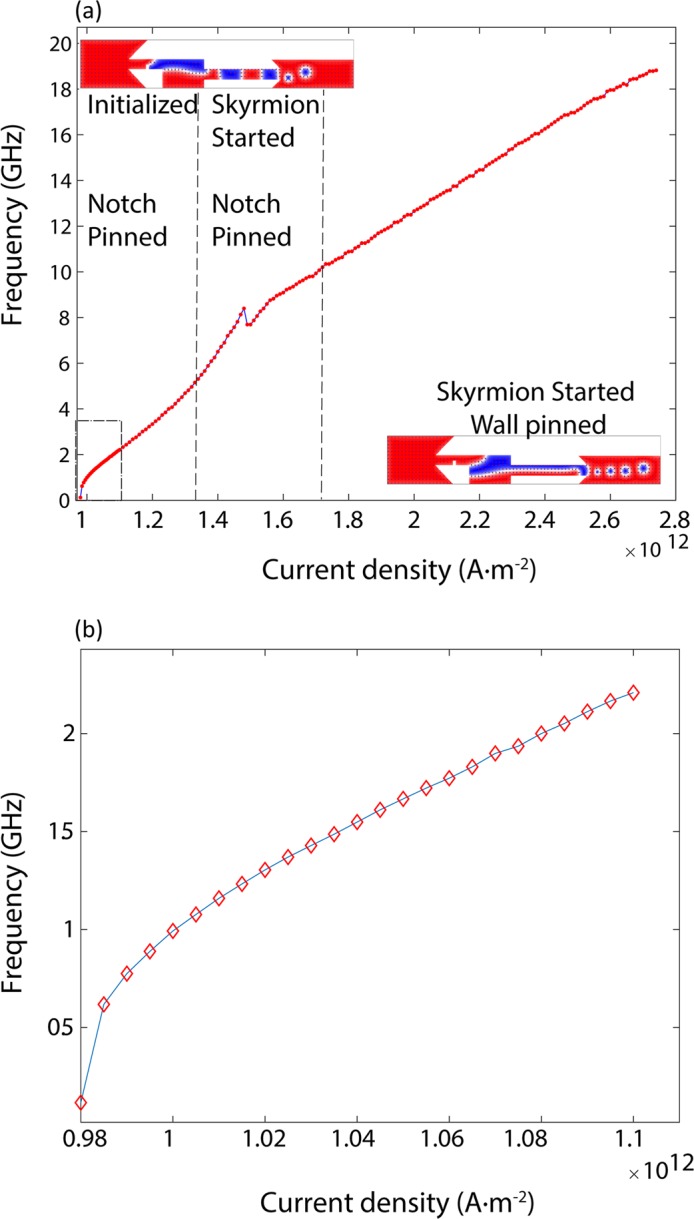
Input current density dependence of skyrmion generation frequency. (**a**) The figure depicts the skyrmion generation rate versus the input current density. Damping coefficient α is 0.3. Skyrmions could be generated for currents from ${\rm{j}}=9.80254\times {10}^{11}\,{\rm{A}}\cdot {{\rm{m}}}^{-2}$ with frequency f = 114 MHz and until ${\rm{j}}=2.74\times {10}^{12}\,{\rm{A}}\cdot {{\rm{m}}}^{-2}$ with frequency f = 18.881 GHz. (**b**) The skyrmion generation frequency shows a square root dependence on the applied current density for low currents and frequencies. This plot corresponds to the dashed rectangle on the lower left-hand corner in (**a**).

The skyrmion generation frequency depends on the timing between steps 4 to 7 of the previous section. If the material, geometric and applied current density parameters are designed such that the domain insertion into the output channel happens faster (as in Fig. 1(b)), the skyrmion generation frequency is higher. Tuning the applied current enables changing this timing. With an increase in the applied current, the domain expansion speed increases. Thus, an increase in the applied current is expected to increase the skyrmion generation frequency. Figure 3 shows the dependence of skyrmion generation frequency on the applied current. The skyrmion generation frequency increases linearly with the applied current except for the low current regime, which is presented in Fig. 3(b).

### Skyrmion generation for lower currents

**Figure 4:**
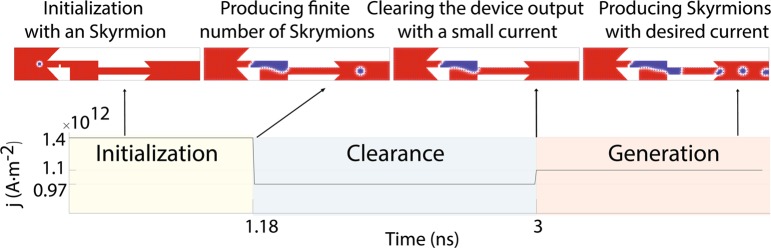
Skyrmion generation for lower currents. Skyrmion generation for currents lower than 1.32 × 10^12^ Am^−2^ can be realized by expanding the domain to the right wall of the reservoir and then using a lower current to generate the skyrmions. Here, the domain has been expanded with a current density equal to $1.40\times {10}^{12}\,{\rm{A}}\cdot {{\rm{m}}}^{-2}$, then the current is lowered to $9.7\times {10}^{11}\,{\rm{A}}\cdot {{\rm{m}}}^{-2}$ to eliminate any remaining skyrmions. Next, a current density equal to $1.1\times {10}^{12}\,{\rm{A}}\cdot {{\rm{m}}}^{-2}$ is applied to generate the low frequency skyrmions.

For applied currents lower than 1.32 × 10^12^ A⋅m^−2^, no skyrmion is generated. This current density is the minimum current density required to push the magnetic domain to the right wall of the reservoir. On the other hand, if we start with a magnetic domain already expanded to the right wall, skyrmions could be generated with lower currents and much lower frequencies. Such an extension of dynamic frequency range could be achieved by applying a higher current to expand the magnetic domain and then using a lower current to generate skyrmions. Figure 4 shows the necessary pulse sequence for this method. In this method, a typical current, e.g. 1.4 × 10^12^ A⋅m^−2^, is applied to initialize the domain within the device. After at least 1.89 ns, we turn the current down to 0.97 × 10^12^ A⋅m^−2^. This current is large enough to clear the output channel and the output track and small enough not to produce additional skyrmions. After this startup phase, one can apply slightly higher currents to reach lower frequencies. We could reach frequencies as low as 114 MHz by a current density of 9.80254 × 10^11^ A⋅m^−2^.

### The effect of Gilbert damping parameter

**Figure 5:**
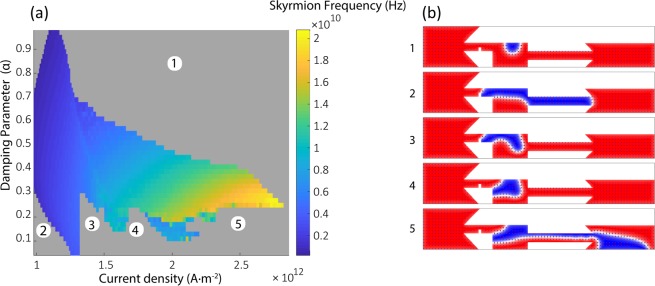
Skyrmion generation frequency based on input current and Gilbert damping coefficient. (**a**) Dependence of skyrmion generation frequency on the applied current density and the damping parameter α. Colorbar indicates the skyrmion generation frequency. Gray regions indicate cases where periodic skyrmions cannot be generated (either no or finite number of skyrmions are generated). (**b**) The domain profiles corresponding to the cases numbered in (**a**) indicate the origin of domain breakdown or generation of finite number of skyrmions.

The applied current density determines the skyrmion generation frequency as discussed previously. In addition, the key material properties such as damping parameter may alter skyrmion frequency. Keeping every parameter constant, the skyrmion generation frequency increases with an increase in the applied current. Figure 5(a) indicates that with higher current densities, one may generally increase skyrmion generation rate.

The damping coefficient α is a parameter that changes the skyrmion generation conditions. Figure 5 shows the effect of damping parameter along with the applied current on skyrmion generation frequency. In the gray regions, either no or finite number of skyrmions are generated. There are different cases where this behavior is observed. These observations are labeled on Fig. 5(a) and their corresponding cases are presented on Fig. 5(b).

In region 1, the magnetic domain moves to the right within the reservoir and vanishes. We see here that an increase in the damping coefficient results in a smaller threshold value and the domain depins with smaller currents. Higher α values result in lower skyrmion frequencies for a constant applied current.

Region 2 shows the case for the smaller and larger α values. Here, the smallest current needed for generating skyrmions depends on the α value. In this case, the domain expands into the output channel but pinching does not happen and no skyrmions are produced.

Regions 3 and 4 reveal an interesting dependency of thresholds on the alpha value. In region 3, the left DW is pinned to the notch but the applied current is not large enough to bring the DW to the right reservoir wall. In region 4, the situation is similar with the domain pinned to the left wall. In addition to the pinning thresholds for the notch and the right wall, different thresholds exist for the domain to reach the right reservoir wall. In region 5, the current threshold for the domain expansion is so low that the magnetic domain expands into the output part.

### Skyrmion energy analysis

For the geometry presented in Fig. 1, one needs to overcome several energy barriers to produce a skyrmion. These energy barriers include the energy required for the magnetic domain to fill the upper part of the reservoir region, the notch energy barrier, the magnetic domain expansion energy, the pinching energy of the magnetic domain, and the skyrmion nucleation energy (Supplementary Movie S8). For information processing beyond Moore’s Law, each information carrier must consume as little energy as thermodynamically feasible. Such energy costs have been defined by Landauer’s limit as low as k_B_Tln(2), although vast majority of the information processing devices even in research settings use many orders of magnitude more energy per bit. Since skyrmions consist of nanoscale spin packets, their formation energy is intrinsically low and near Landauer’s limit. Here, we are interested in the skyrmion generation energy. The total energy before generation of the first skyrmion is 1.12 × 10^−19^ Joules. This energy is needed to push the magnetic domain to the left wall. Having initialized the reservoir domain, the skyrmions could then be generated. The energy costs for each skyrmion is 1.94 × 10^−19^ Joules. This energy is around 67.5 × k_B_Tln(2), where T = 300 K. Therefore, skyrmions might help reach room temperature information processing near Landauer fundamental energy limit for a single bit. This illustrates that the skyrmions have the potential for being very low energy information carriers. Skyrmions could thus offer opportunities for very low energy information processing. Furthermore, this study suggests a path towards eliminating Joule heating significantly by substituting Cobalt layer with a magnetic insulator that can support the skyrmions with the appropriate damping and DMI constants and replacing spin-polarized charge current bias with a pure spin current bias.

In a recent model for skyrmion energy^53^, the equilibrium energy of the skyrmion is E = kAd where A is the exchange interaction coefficient, d is the material thickness and k is a constant for the type of skyrmion generated. For the device with d = 1 nm, A = 15 pJ, and k = 1.94 × 10^−19^/(15 × 10^−12^ × 1 × 10^−9^) = 12.9. Increasing the thickness of the material to 4 nm, changes the skyrmion energy to 7.76 × 10^−19^ Joules. Thus, preserving the value of k = 12.9. Changing the thickness of the material slightly lowers the skyrmion generation frequency as well (Supplementary Fig. SF1). The device operation is robust to changing thickness.

### Room temperature thermal effects

The models presented in this study are for 1 nm thick nanotrack generators. These devices are subject to finite temperature effects and Joule heating. After modeling the device operation with room temperature effects, we found that increasing the cobalt thickness towards 4 nm for the same geometry, yields increased robustness against stochastic temperature effects. (see for 4 nm thick cobalt room temperature operation: Supplementary Movie 8). We found that the skyrmion generators with thicker cobalt layers operate similarly even above room temperature.

### Joule heating effects on temperature stability

Since skyrmions are stabilized along metallic bilayer interfaces, Joule heating is inevitable while passing charge current along these layers. Our energy balance model indicates that for 4 nm thick nanotracks, with charge current density range used in this study, the skyrmion generators would exhibit an equilibrium temperature difference with the substrate given that the device is on a thermally conductive substrate. In our model, heat is generated due to resistive dissipation and given off by thermal conduction to the substrate, thermal radiation, and convection (see the supplementary materials). Since the heat capacity of the substrate is much higher than that of the nanotrack, the heat transfer from the nanotrack does not effectively change the substrate’s temperature. Thus, the temperature difference increases to 336 K due to Joule heating. At this temperature, the heat generated equals the heat dissipated, resulting in a stable temperature. Geometric expansion is negligible at this temperature (For the calculations refer to supplementary materials).

## Conclusion

Here we showed that periodic skyrmion generation is possible with the presented geometry and with realistic material and device parameters in absence of external magnetic field. This device can generate periodic skyrmions with a constant spin-polarized current. The skyrmion frequencies generated using the device could be controlled reversibly with current density from 114 MHz to 21 GHz (over more than seven octaves of frequencies). The frequency range depends on the geometry, the magnitude of the applied current as well as material properties including damping, DMI parameter and anisotropy constants. The initialization and frequency tuning procedures presented allow for dynamic and deterministic adjustment of the generated skyrmion frequencies. As the device does not require external magnetic field, it runs with DC and is robust against the cobalt layer thickness variations, the device architectures presented here could enable researchers design skyrmion circuit layouts for classical or quantum information processing, data storage, sensing and on-chip telecommunications. As the formation energy for each skyrmion is on the order of 50 k_B_T, room temperature ultralow power DC-driven signal processing could be achieved at room temperature near Landauer limit.

The proposed device may be used in integrated skyrmionic chips as efficient, nonvolatile skyrmionic clock or signal sources, encoders for racetrack memory as well as single molecule probes for sensing. The linear frequency response of the device over a large frequency range enables the device to be used as a skyrmionic frequency modulator for telecommunication applications. The periodic skyrmions here could also be used for dynamic refreshing of qubits in quantum information processing by matching the skyrmion generation frequency with the qubit decoherence frequency. The parametric phase diagrams presented here provide deterministic limits on the geometric or material property tolerances for experiments. By using this structure and a modified version of it, one may span and encode the entire telecommunication frequency bands all in the same device. These high-frequency applications, together with previous skyrmion works^45^ could constitute a new sub-area of skyrmions: high-frequency dynamic skyrmionics applications. Skyrmions could thus be used in logic, sensing, memory, telecommunications and quantum information processing applications at room temperature as on-chip signal carriers.

### Note added during review

During peer-review, the authors were made aware of a similar skyrmion study^54^ published while ours was under review. Despite the similarities, these two works fundamentally differ in the design, current range, characterization and stability. In that paper^54^, the regions for the current flow are not explicitly presented. Whether current flows into or from the anti-notch is not clear although this is the crucial step for device operation. The disturbances in current flow at the anti-notch were claimed to change the skyrmion generation capability despite limited evidence provided. The appropriate notch size and shape are essential for device operation although their suggested geometry has very limited tolerance for fabrication errors. In our study, we present simulations proving that the notch is not even essential for wideband skyrmion generation. Different from previous studies, we present a skyrmion generator that can be dynamically tuned by pure DC spin-polarized charge currents over seven octaves of frequencies, present detailed open-source analysis of the operational regimes and failure modes, produce an operational phase diagram of the device over experimentally feasible current and Gilbert damping coefficients, perform an energy analysis per skyrmion bit near Landauer limit, and analyze temperature and heating effects. The current density and damping, rather than the notch geometry, are crucial parameters governing skyrmion generation frequency. The currents applied in our paper is an order of magnitude smaller than that in^54^.

## Methods

### Micromagnetic model

The continuum spin model has been used for the micromagnetic simulations. In this model, the energy density is modeled by the following equation:

1 $$\begin{array}{l} {\mathcal E} =[{{\rm{A}}}_{{\rm{ex}}}{(\nabla {\bf{m}}({\bf{r}}))}^{2}-{{\rm{\mu }}}_{0}{{\rm{M}}}_{{\rm{s}}}{\bf{m}}({\bf{r}}).{{\bf{H}}}_{{\rm{external}}}-{{\rm{\mu }}}_{0}{{\rm{M}}}_{{\rm{s}}}{\bf{m}}({\bf{r}}).{{\bf{H}}}_{{\rm{demag}}}-\,{{\rm{K}}}_{{\rm{u}}}({({\bf{m}}({\bf{r}}).{{\bf{e}}}_{{\rm{z}}})}^{2})\\ \,+{{\rm{D}}}_{{\rm{i}}}({{\rm{m}}}_{{\rm{z}}}\nabla .{\bf{m}}({\bf{r}})-({\bf{m}}({\bf{r}}).\nabla ){{\rm{m}}}_{{\rm{z}}})+{{\rm{D}}}_{{\rm{b}}}{\bf{m}}({\bf{r}}).(\nabla \times {\bf{m}}({\bf{r}}))]\end{array}$$

where **m(r)** is the local normalized magnetic moment orientation ${\bf{m}}({\bf{r}})=\frac{{\bf{M}}}{{{\bf{M}}}_{{\bf{s}}}}\,{\rm{with}}\,{\rm{normalized}}\,{\rm{magnitude}}\,|{\bf{m}}({\bf{r}})|=1$, A_ex_ is the exchange stiffness coefficient, μ_0_ is the vacuum magnetic permeability, M_s_ is the saturation magnetization, **H**_external_ is the applied magnetic field, **H**_demag_ is the Demagnetization field, K_u_ is the uniaxial anisotropy constant, **e**_**z**_ is the anisotropy easy axis direction. D_i_ and D_b_ are the interface and bulk Dzyaloshinskii-Moriya interaction energy densities, resulting in Néel-type (hedgehog) and Bloch-type (vortex) skyrmions respectively.

The magnetization dynamics follow the Landau-Lifshitz-Gilbert (LLG) with spin-transfer torque (STT) terms as follows:

2 $$\frac{{\rm{d}}{\bf{m}}}{{\rm{dt}}}={{\rm{\gamma }}}_{{\rm{LL}}}\frac{1}{1+{{\rm{\alpha }}}^{2}}({\bf{m}}\times {\bf{B}}{}_{{\rm{eff}}}+{\rm{\alpha }}({\bf{m}}\,\times ({\bf{m}}\,\times {{\bf{B}}}_{{\rm{eff}}})))+{{\rm{\tau }}}_{{\rm{ZL}}}+{{\rm{\tau }}}_{{\rm{Sl}}}$$

where γ_LL_ is the gyromagnetic ratio for LLG equation, **B**_eff_ is the effective magnetic field, α is the damping coefficient, and τ_ZL_ is the Zhang-Li STT for current in plane (CIP), and τ_Sl_ is the Sloncewski STT term for current perpendicular to plane (CPP) situations.

The Zhang-Li equation accounts for the spin transfer torques arising from the transferred torques of non-equilibrium conduction electrons of a current flowing in-plane in the magnetic layer^55^:

3 $${{\rm{\tau }}}_{{\rm{ZL}}}=\frac{1}{1+{{\rm{\alpha }}}^{2}}[(1+{\rm{\xi }}{\rm{\alpha }}){\bf{m}}\times ({\bf{m}}\times ({\bf{u}}.\nabla ){\bf{m}})+({\rm{\xi }}-{\rm{\alpha }}){\bf{m}}\times ({\bf{u}}.\nabla ){\bf{m}}]$$

Where

4 $${\bf{u}}=\frac{{{\rm{\mu }}}_{{\rm{B}}}{{\rm{\mu }}}_{0}}{2{{\rm{e}}{\rm{\gamma }}}_{0}{{\rm{B}}}_{{\rm{sat}}}(1+{{\rm{\xi }}}^{2})}{\bf{j}}$$

While ξ is the degree for non-adiabaticity for STT, μ_B_ is Bohr magneton, e is the fundamental charge, B_sat_ is the saturation magnetic field, and **j** is the current density vector. For the current flowing in the x direction. ${\bf{j}}={\rm{j}}\,{\hat{{\rm{e}}}}_{{\rm{x}}}$. For the elimination of skyrmion hall effect we suppose ξ = α thus the Zhang-Li term reduces to:

5 $${{\rm{\tau }}}_{{\rm{ZL}}}=\frac{1}{1+{{\rm{\alpha }}}^{2}}\times \frac{{{\rm{\mu }}}_{{\rm{B}}}{{\rm{\mu }}}_{0}{\rm{j}}}{2{e{\rm{\gamma }}}_{0}{{\rm{B}}}_{{\rm{sat}}}}{\bf{m}}\times ({\bf{m}}\times \frac{\partial }{\partial {\rm{x}}}{\bf{m}})$$

which is a damping-like non-adiabatic term.

The Slonczewski term accounts for the STT from a perpendicular current flowing in the nanotrack^56^. This perpendicular current may enter from a layer adjacent to the nanotrack. The Slonczewski STT is:

6 $${{\rm{\tau }}}_{{\rm{Sl}}}={\rm{\beta }}\frac{{\rm{\epsilon }}+{\rm{\alpha }}{{\rm{\epsilon }}}^{\text{'}}}{1+{{\rm{\alpha }}}^{2}}({\bf{m}}\times ({{\bf{m}}}_{{\bf{p}}}\times {\bf{m}}))-{\rm{\beta }}\frac{{{\rm{\epsilon }}}^{\text{'}}-{\rm{\alpha }}{\rm{\epsilon }}}{1+{{\rm{\alpha }}}^{2}}({\bf{m}}\times {{\bf{m}}}_{{\bf{p}}})$$

Where

7 $${\rm{\beta }}=\frac{{{\rm{j}}}_{{\rm{z}}}\hslash }{{{\rm{M}}}_{{\rm{sat}}}{\rm{ed}}}$$

And

8 $${\rm{\epsilon }}=\frac{{\rm{P}}({\rm{r}},{\rm{t}}){{\rm{\Lambda }}}^{2}}{({{\rm{\Lambda }}}^{2}+1)+({{\rm{\Lambda }}}^{2}-1)({\bf{m}}.{{\bf{m}}}_{{\bf{p}}})}$$

where j_z_ is the current density along the z axis, d is the magnetic layer thickness, **m**_**p**_ is the fixed-layer magnetization, P the spin polarization, the Slonczewski Λ parameter characterizes the spacer layer, and ϵ′ is the secondary spin-torque parameter. It should be noted that in the current study and other similar studies spacer layer is nonexistent. Here, we only consider the current in-plane case.

For finite temperature simulations, the thermal noise is modeled according to Brown^57^

9 $${\overrightarrow{{\rm{B}}}}_{{\rm{therm}}}=\overrightarrow{{\rm{\eta }}}({\rm{step}})\sqrt{\frac{2{{\rm{\mu }}}_{0}{{\rm{\alpha }}{\rm{k}}}_{{\rm{B}}}{\rm{T}}}{{{\rm{B}}}_{{\rm{sat}}}{{\rm{\gamma }}}_{{\rm{LL}}}{\rm{\Delta }}{\rm{V}}{\rm{\Delta }}{\rm{t}}}}$$

Where α is the damping parameter, k_B_ the Boltzmann constant, T is e temperature in kelvin, B_sat_ the saturation magnetization expressed in Tesla, γ_LL_ the gyromagnetic ratio, ΔV the cell volume, Δt the time step and $\overrightarrow{{\rm{\eta }}}({\rm{step}})$ a random vector from a standard normal distribution whose value is changed after every time step.

The electric current flows from right to left, i.e. electrons flow from left to right since the spin transfer torque effects in the direction of the charge current flow (Supplementary Fig. SF2). All electric current densities reported in this study are calculated for the point where they enter the leftmost part of the device with 100 nm width. The current density at other parts is this current density multiplied by the ratio of the widths (${{\bf{j}}}_{{\rm{x}}}=\frac{100{\rm{nm}}}{{{\rm{w}}}_{{\rm{x}}}}\times {{\bf{j}}}_{0}$) i.e the current density in the narrow channel with w = 20 nm is five times larger than the initial current density.

The change in the thickness would slightly shift skyrmion generation frequency (Supplementary Fig. SF3) as demagnetizing field changes.

### Skyrmion modeling

The skyrmion is a spin arrangement having the topological protection because the invariant

10 $${\rm{N}}=\frac{1}{4{\rm{\pi }}}\int {{\rm{d}}}^{2}{\rm{r}}{\bf{m}}.(\frac{\partial {\bf{m}}}{\partial {\rm{x}}}\times \frac{\partial {\bf{m}}}{\partial {\rm{y}}})$$

is an integer. Because of the discrete nature of the lattice, this topological protection is not ideal and may be broken with enough force. This breakdown happens when the skyrmion is pushed into a constriction narrower than its width.

### Material parameters

For the simulation parameters, we used the typical cobalt characteristics. The uniaxial anisotropy is K_u_ = 0.8 MJ⋅m^−3^, The Exchange energy density is A_ex_ = 15 pJ⋅m^−3^, the interface Dzialoshinskii-Moriya interaction is D = 3.5 mJ⋅m^−2^, saturation magnetization M_s_ = 580 kA⋅m^−1^, and the non-adiabatic spin transfer coefficient is set equal to alpha to prevent the skyrmion Hall effect. 1 nm mesh size was used for simulations for finer spatial resolution.

The simulations have been implemented with GPU enabled Mumax3 on high-performance computational clusters. The skyrmions were detected by image averaging of a region of interest. The skyrmion frequency range was then calculated using a peak detection algorithm on the averaged image data. All MATLAB and Mumax3 simulation^41^, processing and post-processing scripts are provided as fully open-source in the supplementary materials.

## Supplementary information

Supplementary Materials

Supplementary Movie 1

Supplementary Movie 2

Supplementary Movie 3

Supplementary Movie 4

Supplementary Movie 5

Supplementary Movie 6

Supplementary Movie 7

Supplementary Movie 8

Supplementary Movie 9

Supplementary Movie 10

Supplementary Movie 11

Supplementary Movie 12

Supplementary Movie 13
