# Supplementary material for: Direct current-tunable MHz to multi-GHz skyrmion generation and control
Source: Sci Rep. 2019 Jul 1;9:9496. doi: 10.1038/s41598-019-45972-9 (PMC6603187; doi:10.1038/s41598-019-45972-9)
Supplement: Supplementary file 1 — Supplementary Materials [file 41598_2019_45972_MOESM1_ESM.pdf]

Supplementary Materials for "Direct current-tunable MHz to multi-GHz skyrmion generation and control"

Arash Mousavi Cheghabouri[1] & Mehmet C. Onbasli[1,*]

[1]Department of Electrical and Electronics Engineering, Koç University, Sarıyer, 34450 Istanbul, Turkey.
* Corresponding Author: monbasli@ku.edu.tr

Table of contents

Contents

Bloch skyrmion results 2
Current Flow 2
Heating and temperature effects 3
The effect of thickness on skyrmion generation. 4
Different Geometries 5
Movies 5
Codes 6
  Simulation 6
  Processing 8

Bloch skyrmion results

With a material with bulk DMI, Bloch type skyrmions are stabilized instead of Néel type. We found that the dynamics is similar for the Bloch type skyrmions. Supplementary Figure 1 shows skyrmion generation frequencies for both Néel and Bloch type skyrmions.

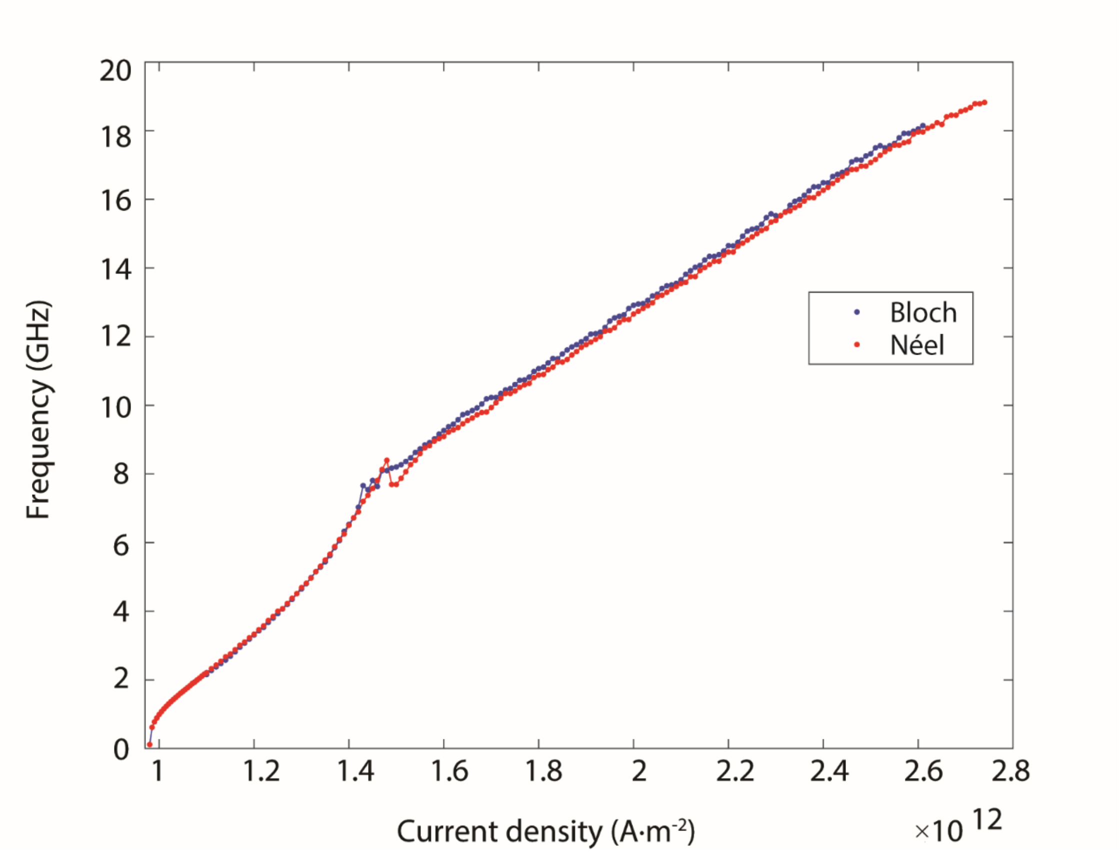

Supplementary Figure SF1. Bloch and Néel skyrmion generation rate. The generation rate for both skyrmion types are close.

The difference between the generation rate for Bloch and Néel skyrmions may be due to detection error resulting from the difference in magnetization profiles for these two skyrmion types.

Current Flow

The spin-polarized charge current is injected in the current in plane (CIP) setting. The transferred torques follow the Zhang-Li STT formula. The domain walls move in the direction of the electron flow and opposite to the direction of the current flow. The current density depends on the cross-section area of the nanotrack. Since the thickness of the nanotrack is constant throughout its length, the current density for each region depends on the width of the region. To model this dependence, the device is split into the different regions. Supplementary Figure 2 shows the device and the region names.

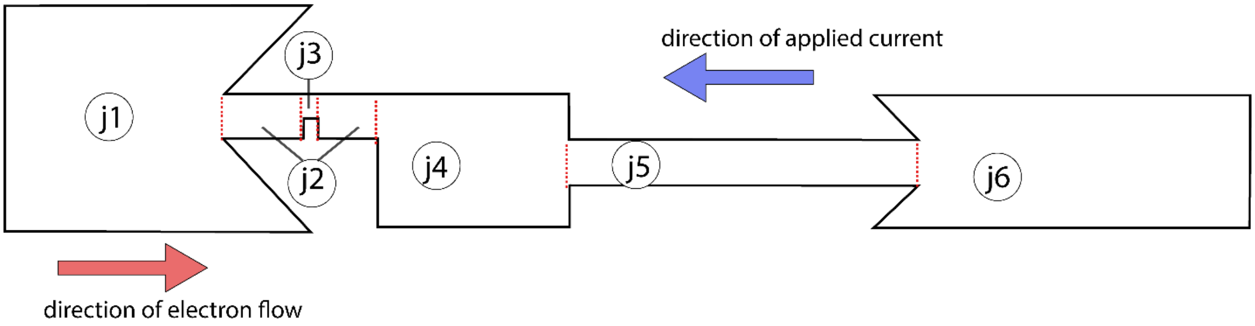

Supplementary Figure SF2. Different regions for current flow. Each region is labeled according to the label used in the simulation scripts.

The naming of the regions on the figure follows the variable names used in the simulation script for each region. Since the current does not change abruptly from a region to another, the narrower regions can be extended in both sides for some nanometers.

Heating and temperature effects

For further investigation into finite temperature effects, one may evaluate the input and output dissipative powers for the device. The source for the dissipative power in the device is the Joule heating from current. If the current passes through a bilayer stack of Pt and Co, we may write the Joule heating as:

$$P_{in} = P_{Pt} + P_{Co} = I_{Co}^2 R_{Co} + I_{Pt}^2 R_{Pt} = I^2 \times R_{stack}$$

Where $I = j \times w \times d$ and $\frac{1}{R_{stack}} = \frac{1}{R_{Pt}} + \frac{1}{R_{Co}}$. For a patterned Co/Pt bilayer with the present device's geometry, $R_{stack} = 1677\ \Omega$ and the input power is 9 mW for $j = 2.9 \times 10^{12}\,A \cdot m^{-2}$. The system may lose energy by thermal radiation, thermal convection and thermal conduction. The energy loss by radiation and convection are negligible compared to conduction. Supposing the flat side of the nanotrack is in contact with a heat sink maintaining a constant temperature (i.e. sufficiently large thermal reservoir like a substrate) and considering the thermal conductivity of Pt and Co, the heat loss of the device is:

$$P_{out} = \frac{k\Delta TA}{d}$$

Where ΔT is the temperature difference between the nanotrack and the heat sink. Solving for the temperature difference while $P_{out} = P_{in}$ results in $\Delta T = 35.6$°C. The skyrmion generator achieves dynamic thermal equilibrium once the temperature difference between the device and the thermal reservoir reaches 35.6°C. The device would thus equilibrate at $T = 336$ K for a thermal reservoir at room temperature (substrate at T_{room} = 300 K). Since Supplementary Movie 8 shows robust skyrmion generation at 350 K, we demonstrate that the thermally equilibrated device can work as designed. Thus, a set of realistic parameters leads to efficient prevention of the device heat up. Some skyrmions are lost which

will result in a decrease in information content and skyrmion generation frequency, but the working of the device does not get interrupted.

The effect of thickness on skyrmion generation

The skyrmion generation rate depends on the magnetization dynamics resulted from the STT terms. The magnetization dynamics follows the LLG equation equipped with the STT terms[7]:

$$\frac{d\mathbf{m}}{dt} = \gamma_{LL}\frac{1}{1+\alpha^2}\left(\mathbf{m} \times \mathbf{B}_{\text{eff}} + \alpha\left(\mathbf{m} \times (\mathbf{m} \times \mathbf{B}_{\text{eff}})\right)\right) + \tau_{STT}$$

In this equation, when the ratio of STT torques to the LLG dynamic term (the sum of the precession and damping terms on the right hand side of the equation) decreases, the magnetization dynamics due to these STT terms decreases too. With a thicker nanotrack, the demagnetization field decreases. This decrease results in an increase in B_{eff}, which in turn increases the LLG precession and damping terms, while the STT terms remain constant. Thus, the τ_{STT} term, which contains the torques that move the domain wall, decreases with respect to the first two terms (precession and damping). With higher frequencies, there are more skyrmions present in the device. Supplementary Figure 3 shows the skyrmion generation frequency vs. applied current density for the 1 nm and 4 nm nanotracks.

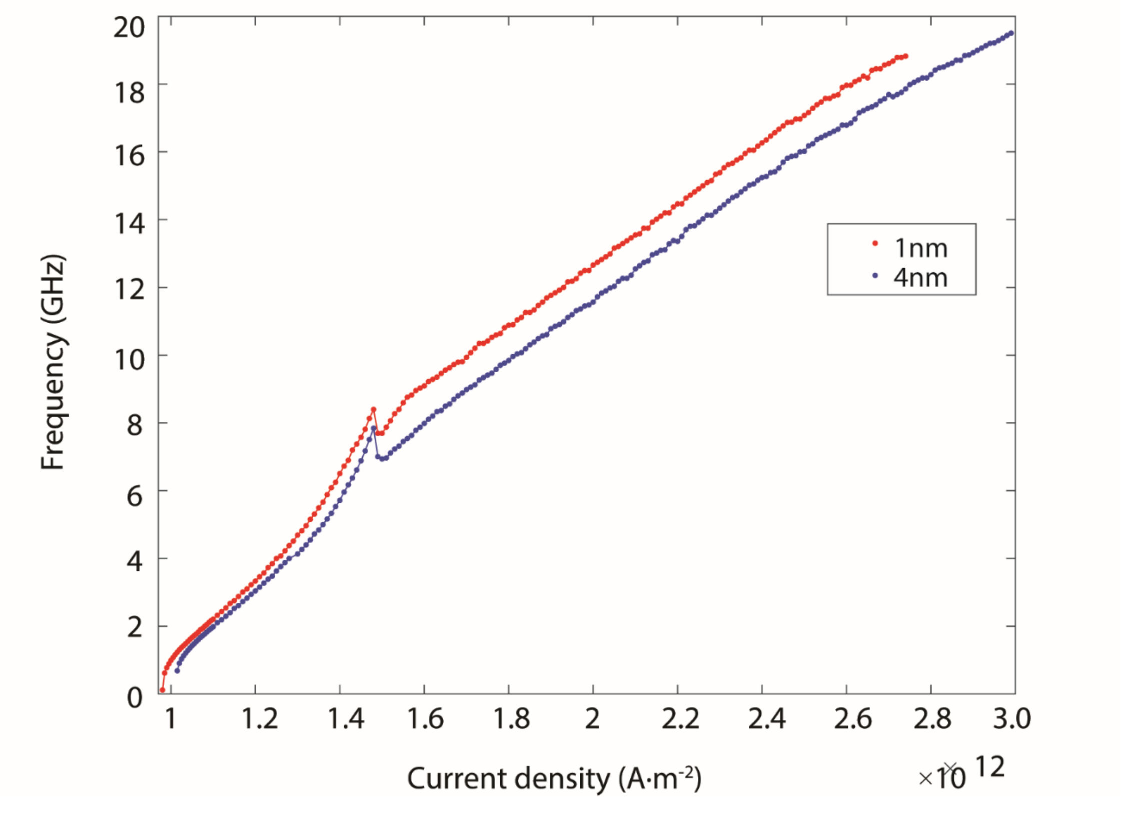

Supplementary Figure SF3. Skyrmion generation frequency for 1 nm and 4 nm nanotracks. The generated skyrmion frequency is lower for the thicker nanotrack and the skyrmion generation is feasible for higher currents.

Different Geometries

While the proposed geometry covers all working modes of the proposed device, deviating from this geometry is possible given that following guidelines are observed.

1- There must exist a magnetic domain.
2- The left wall of the magnetic domain must be pinned.
3- There must be a constriction for pinching out an extended part of the domain.
4- There must be an output track to stabilize the pinched domain walls into skyrmions.

Thus, one may be interested in the simplification of the geometry in favor of ease of fabrication. Here we provided some simplified versions of the proposed device. Movies 10 to 13 show different geometries of the device.

Movies

Supplementary Movie S1: A rectangular notch acts as a potential barrier to the magnetic domain. The height of this potential barrier is different for different wall chiralities. For the right wall, the potential barrier is smaller than the left wall. With a proper current that can surpass the right wall barrier and not the left wall barrier, right wall passes the notch but the left wall sticks to the notch. This entails in the expansion of the magnetic domain.

Supplementary Movie S2: With a current small enough that cannot push the right wall from the notch. The current pushes the left wall into the right wall making the magnetic domain smaller which eventually results in the annihilation of the magnetic domain.

Supplementary Movie S3: With a current bigger than the left wall passing current, both walls pass the notch.

Supplementary Movie S4: The generator working with the normal condition when the current is smaller than the notch depinning current.

Supplementary Movie S5: When the current is not big enough to expand the magnetic domain into the right wall of the reservoir.

Supplementary Movie S6: When the current is bigger than the notch depinning current, the left wall departs from the notch, but the left wall of the reservoir acts as an anti-notch that pins the wall like a notch.

Supplementary Movie S7: When the current is bigger than the left wall (anti-notch) depinning current, the magnetic domain will not pin to any site and vanishes into the right wall of the reservoir.

Supplementary Movie S8: The 4 nm-thick nanotrack at T = 350 K.

Supplementary Movie S9: The input track is eliminated. Thus, the operation of the device may be started with a domain wall instead of a skyrmion.

Supplementary Movie S10: The Supplementary Movie 9 geometry without a notch.

Supplementary Movie S11: The proposed device without a notch.

Supplementary Movie S12: The proposed device without a notch and with rectangular vertices.

Supplementary Movie S13: The working of the device with bulk DMI and Bloch type skyrmions.

Codes

The simulation and data processing have several steps which we explain shortly below. The simulation is carried out using Mumax3, a GPU accelerated micromagnetic simulation framework.

Simulation

A simulation script for a typical simulation session is as follows. This script simulates and saves the results of the simulations in the .ovf format for every 0.01 ns.

Simulation.mx3:

```
SetMesh(550, 100, 1, 1e-09, 1e-09, 1e-09, 0, 0, 0);
setgeom(imageShape("generator.png"));
alpha = 0.3;
xi = 0.3;
Aex = 15e-12;
Ku1 = 0.8e6;
Msat = 580000;
Dind = 3.5e-3;
m = uniform(0 , 0 , 1);
anisU = vector(0, 0, 1);
defregion(10 , rect(70e-9 , 70e-9).transl(-200e-9 , 0, 0));
m.setregion(10 , neelskyrmion(1 , -1).transl(-200e-9,0, 0));
JJ:=-1.40e+12;
Jd1:=100/100;
Jd2:=100/60;
Jd3 := 100/20;
Jd4 := 100/12;
Jd5:=100/20;
Jd6:=100/60;
defregion(1 , imageshape("j1.png"));
defregion(2 , imageshape("j2.png"));
defregion(3 , imageshape("j3.png"));
defregion(4 , imageshape("j4.png"));
defregion(5 , imageshape("j5.png"));
defregion(6 , imageshape("j6.png"));
j.setregion(1 , vector(JJ*Jd1 , 0 , 0));
j.setregion(2 , vector(JJ*Jd2 , 0 , 0));
j.setregion(3 , vector(JJ*Jd3 , 0 , 0));
j.setregion(4 , vector(JJ*Jd4 , 0 , 0));
j.setregion(5 , vector(JJ*Jd5 , 0 , 0));
j.setregion(6 , vector(JJ*Jd6 , 0 , 0));
tableAdd(E_anis);
tableAdd(E_total);
tableAdd(E_exch);
Pol =1;
relax();
run(2e-9);
autosave(m, 0.01e-9);
```

```
tableautosave(0.01e-9);
run(3e-9);
```

After the simulation we need to process the .ovf files. For this end, we use the OOMMF conversion utility for converting .ovf files into .bmp files. The below code snippet uses avf2ppm utility of the OOMMF package to convert the ovf files to bmp files. For this code snippet the OOMMF package is installed in “c:\oommf\” and the setting file is “C:\settings\setting.config”. One should change these paths according to their computer.

Tobmp.txt:

```
tclsh c:\oommf\oommf.tcl avf2ppm -f -format b24 -config
"C:\supplementary\settings.config" *.ovf
```

The setting file for the simulation is:

Settings.config:

```
# FILE: setting.config           -*-Mode: tcl-*-
#
# mmDisp configuration.  This file must be valid Tcl code.

# This file should only be sourced from within another Tcl application.
# Check to make sure we aren't at the top application level
if {[string match [info script] $argv0]} {
    error "'[info script]' must be evaluated by an mmdisp-style application"
}

# Plot configuration array
array set plot_config {
   arrow,autosample          1
   arrow,colorcount          256
   arrow,colormap            Red-Black-Blue
   arrow,colorphase          0
   arrow,colorreverse        0
   arrow,quantity            z
   arrow,size                1
   arrow,status              1
   arrow,subsample           7
   arrow,antialias                     1
   misc,background           white
   misc,boundarycolor        black
   misc,boundarywidth        1
   misc,datascale            1
   misc,defaultwindowheight  480
   misc,defaultwindowwidth   640
   misc,drawboundary         1
   misc,margin               0
   misc,rotation             0
   misc,width                5000
   misc,zoom                 3
   pixel,autosample          1
```

```
    pixel,colorcount           256
    pixel,colormap             Blue-White-Red
    pixel,colorphase           0
    pixel,colorreverse         0
    pixel,opaque               1
    pixel,quantity             z
    pixel,size                 0.1
    pixel,status               1
    pixel,subsample            0
    viewaxis                   +z
    viewaxis,xarrowspan        {}
    viewaxis,xpixelspan        {}
    viewaxis,yarrowspan        {}
    viewaxis,ypixelspan        {}
    viewaxis,zarrowspan        1.000e-9
    viewaxis,zpixelspan        1.000e-9
}

array set print_config {
    croptoview                 1
    hpos                       center
    lmargin                    1.0
    orient                     landscape
    paper                      letter
    pheight                    1.239
    pwidth                     6.0
    tmargin                    1.0
    units                      in
    vpos                       center
}
```

Processing

For a better understanding of the course of the simulation and saving the disk drive space we can convert the image sequence into a movie. This is done in MATLAB.

Makemovie.m:

```
%This script produces a movie out of the simulation images
path = 'C:\simulation_path';
d = dir([path  '/*.bmp']);
siz = size(d);
outVideo = VideoWriter([path '/movie.avi']);
outVideo.FrameRate = 25;
open(outVideo);
counter = 1;
for i = 1:siz
    if(length(d(i).name) == 11)
        kk(counter , :) = d(i).name;
    end
    counter = counter + 1;
```

```
end
kk = sortrows(kk);
siz = size(kk);
temp = zeros(1 , siz(1));
for i = 1:siz(1)
    if strcmp(kk(i , 1), 'm')
        img =  imread([path '/' kk(i , :)]);
        writeVideo(outVideo,img);
    end


end
close(outVideo);
clear kk outVideo
```

This code produces a movie from the converted images of the simulation.

This movie may then be disintegrated into images and processed to extract the skyrmion generation rate.

Process.m:

```
path = 'C:\simulation_path';
v = VideoReader([path '/movie.avi']);
i = 1;
while v.hasFrame
    img = v.readFrame;
    img = img(130:end , 1500:1503 , 3);
    temp(i) = sum(sum(img>250));
    i = i+1;
end
save([path '/data.mat'])
```

we then can extract the frequency of this image sequence using peak detection:

frequencyextract.m:

```
filename = 'C:\simulation_path\data.mat';
fsfactor = 0.01;
conv1 = 3;
offset = 3;
endset = 3;
wind = ones(1 , conv1)/conv1;
load(filename);
fs = fsfactor*1e-9;
th = 30;
data1= temp;
data = conv(data1 , wind);
dsiz = size(data);
data(data<th) = 0;
[y , mx] = findpeaks(data,'MinPeakWidth',1);
if ~isempty(mx)
    freqdata = 1e9/(fsfactor*mean(diff(mx(offset:end-endset))));
```

```
else
    freqdata = nan;
end
```

freqdata is the extracted skyrmion frequency for the simulation.

1 Vansteenkiste, A. *et al.* The design and verification of MuMax3. *AIP advances* **4**, 107133 (2014).
